# Three Distinct Domains Contribute to Nuclear Transport of Murine Foxp3

**DOI:** 10.1371/journal.pone.0007890

**Published:** 2009-11-18

**Authors:** Wayne W. Hancock, Engin Özkaynak

**Affiliations:** 1 Division of Transplant Immunology, Department of Pathology and Laboratory Medicine, Children's Hospital of Philadelphia and the University of Pennsylvania, Philadelphia, Pennsylvania, United States of America; 2 Department of Pathology and Laboratory Medicine, Children's Hospital of Philadelphia and the University of Pennsylvania, Philadelphia, Pennsylvania, United States of America; New York University School of Medicine, United States of America

## Abstract

Foxp3, a 47-kDa transcription factor, is necessary for the function of CD4+CD25+ regulatory T cells (Tregs), with an essential role in the control of self-reactive T cells and in preventing autoimmunity. Activation of Tregs by TCR engagement results in upregulation of Foxp3 expression, followed by its rapid nuclear transport and binding to chromatin. Here, we identify three distinct Foxp3 domains that contribute to nuclear transport. The first domain (Domain 1) comprises the C-terminal 12 amino acids. The second domain (Domain 2) is located immediately N-terminal to the forkhead domain (FHD), recently reported to be a binding site for the runt-related transcription factor 1/acute myeloid leukemia 1 (Runx1/AML1). The third domain (Domain 3) is located within the N-terminal first 51 amino acids. Unlike the known nuclear localization signals (NLSs), none of these three regions are rich in basic residues and do not bear any similarity to known monopartite or bipartite NLSs that have one or more clusters of basic amino acids. The basic arginine-lysine-lysine-arginine (RKKR) sequence, located 12-aa from the C-terminal end of Foxp3 was previously reported to be a nuclear localization signal (NLS) for several proteins, including for a GFP-Foxp3 hybrid. Evidence is provided here that in the full-length native Foxp3 RKKR does not function as an NLS. The data reported in this study indicates that Foxp3 achieves nuclear transport by binding to other nuclear factors and co-transporting with them to the nucleus.

## Introduction

CD4+CD25+ regulatory T cells (Tregs) play a critical role in establishing immune tolerance and in prevention of autoimmunity. Foxp3, a DNA-binding protein expressed by natural Tregs, is necessary for Treg function. Ectopic expression of Foxp3 in CD4+CD25- cells is sufficient for their conversion to a Treg phenotype [1], [2]. In mice, a frame-shift mutation in the Foxp3 forkhead domain (FHD) results in the loss of the DNA-binding residues, leading to the Scurfy phenotype and lethal autoimmunity [1], [3]. In humans, multiple mutations in the Foxp3 gene can lead to IPEX (immunodysregulation, polyendocrinopathy, enteropathy, X-linked), a syndrome that is similar to Scurfy, with multi-organ lymphocytic infiltration, lympadenopathy, exfoliative dermatitis, hepatosplenomegaly and autoantibodies [4]–[6].

Like other members of the FoxP subfamily, Foxp3 has zinc-finger and leucine-zipper domains. Foxp3 can form homo-dimers and tetramers, and can associate with Foxp1 [7]. The leucine-zipper domain of Foxp3 plays an essential role in the formation of higher order structures [7]. Foxp3 can exist as part of a large functional complex of ∼600 kDa, together with histone deacetylases, histone acetyltransferases and Runx1/AML1) [8], [9]. Its association with several transcription factors has been reported, including retinoid-related orphan receptors -α and -γt (ROR-α and ROR-γt) [10], [11], nuclear factor of activated T cells (NFAT), nuclear factor of kappa light polypeptide gene enhancer in B-cells (NF-κB) and Activator protein (AP-1) [12]–[14]. The expression and function of Foxp3 is regulated by acetylation [8]. Foxp3 is processed by proteolytic cleavage events at two RXXR motifs (^48^RDLR^51^ and ^414^RKKR^417^), resulting in different forms that are functionally distinct [15].

Binding of Foxp3 to different promoters can lead to acetylation of histones at some promoters (GITR, CD25 and CTLA-4) and to deacetylation at others (IL-2 and IFN-γ) [16]. Foxp3 is a multifunctional protein, and consistent with its opposite effects on promoter acetylation, it functions as a transcriptional repressor or an activator [13], [16].

Although Foxp3 has been intensively studied, little is known about how it translocates from cytoplasm into the nucleus. The mechanism of Foxp3 nuclear import was thus the focus of this study. WT and Foxp3 mutants were retrovirally expressed in CD4+ cells and after transduction, the rate of Foxp3 nuclear translocation was determined by Western blot analysis of the subcellular fractions. The results show that three separate regions contribute to nuclear transport of Foxp3.

## Results

### The Basic RKKR Sequence Does Not Function as a Classic NLS in the Full-Length Foxp3

**Figure 1:**
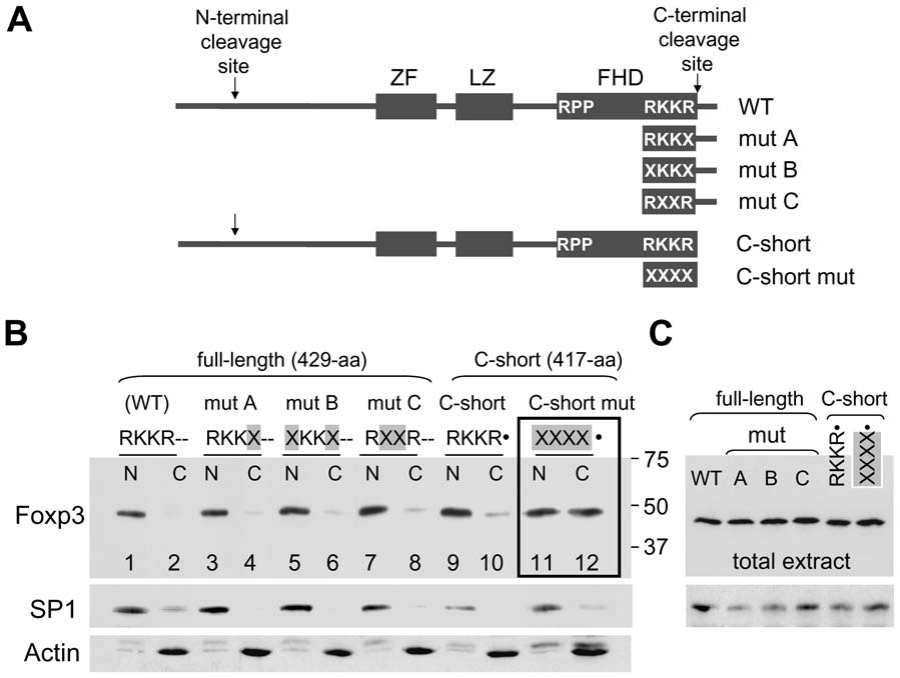
Comparison of the nuclear translocation abilities of WT Foxp3 and Foxp3 mutants. **A.** Schematic diagram showing the approximate locations of the zinc-finger (ZF), leucine-zipper (LZ) and the forkhead domain (FHD) in the full-length WT mouse Foxp3. Vertical arrows indicate sites of proteolytic cleavage. The amino acid sequences RPP and RKKR shown in the schematic diagrams represent the first and the last residues of the FHD. Mutant residues in mut A, mut B, mut C, and C-short mutant Foxp3 are indicated with an X. The lower diagram shows the structure of C-short Foxp3 lacking the terminal 12-aa tail. This engineered Foxp3 mimics the natural C-cleaved form of the protein, a result of proteolytic processing at ^414^RKKR↓S^418^. **B.** Western blot showing nuclear and cytoplasmic distribution of WT Foxp3 (RKKR--), mut A (RKKX--), mut B (XKKX--), mut C (RXXR--), C-short Foxp3 (RKKR•) and C-short Foxp3 mutant (XXXX•). Lanes 11 and 12 (inside the frame), corresponding to C-short mutant (XXXX•), reveal an impaired rate of nuclear translocation. CD4+ cells were harvested 24 hr after retroviral transduction and subcellular fractions obtained. Mutant residues on all the Western blot figures are shown with X and highlighted in grey. • indicates engineered ends and double dashes (--) indicate the 12-aa tail. Following treatment with the Foxp3 mAbFJK-16s, the membrane was treated with SP1 and Actin antibodies to determine the efficiency of subcellular fractionation. The second species migrating slightly slower then Actin in lanes 1-12 is Foxp3. Foxp3 signal was generated along with the Actin signal due to previous treatment of the membrane with Foxp3 antibody. N, nuclear extract; C, cytoplasmic extract. The N and C designations are used in all the figures. **C.** Western blot showing equal Foxp3 expression in total extracts obtained from samples shown in B. SP1 signal was used to determine loading.

RKKR was reported to be a NLS for several proteins, including for the GFP-Foxp3 hybrid [17]–[20]. To determine whether it functions as a NLS in native Foxp3, several constructs were made in which single or multiple residues of ^414^RKKR^417^ were replaced with unrelated amino acids. The nuclear transport properties of WT and mutant Foxp3s were then determined in CD4+ cells using retroviral expression, followed by Western blot analysis of the subcellular fractions. The replacement of the second arginine in the first mutant with an unrelated amino acid did not alter its nuclear transport kinetics (mutant A; RKKR to RKKX; replaced residues underlined) (Fig. 1). Subsequent replacement of both of the arginines (RKKR to XKKX) (mutant B) or both of the lysines (RKKR to RXXR) (mutant C) similarly did not have any effect, indicating RKKR does not function as a NLS in the full-length protein (Fig. 1).

Foxp3 was recently shown to be processed immediately after two RXXR motifs [15]. N-terminal cleavage of Foxp3 after RDLR (^48^RDLR↓S^52^) generates N-cleaved Foxp3, C-terminal cleavage after RKKR (^414^RKKR↓S^418^) generates C-cleaved Foxp3, and cleavage at both ends generates N&C-cleaved (double-cleaved) form of the protein. The C-cleaved form (417-aa), ending with RKKR, is only 12-aa shorter than the full-length 429-aa Foxp3. To determine whether RKKR in short C-cleaved Foxp3 serves as a NLS, a construct that encodes Foxp3 mimicking the C-cleaved form (missing residues 418–429) was generated (referred to as C-short, designated as RKKR• where • indicates engineered stop codon). In a separate construct, the terminal residues of C-short (RKKR•) were replaced with unrelated amino acids (XXXX•). Retroviral expression showed, while the C-short Foxp3 (RKKR•) could transport to the nucleus at a similar rate as the full-length protein, the C-short mutant (XXXX•) had a slower nuclear translocation rate (Fig. 1). This data contrasts the data obtained from full-length Foxp3 mutants where replacement of RKKR residues were found not to alter the kinetics of nuclear transport (Fig. 1, mutants A–C). The results show, unlike in full-length Foxp3, the terminal RKKR in C-short Foxp3 can function as a NLS. A likely possibility is that in the full-length Foxp3 RKKR is masked by the 12-aa tail whereas in C-short Foxp3 RKKR is exposed and recognized by the NLS-mediated nuclear import machinery. However, a biological role for RKKR as a NLS is unlikely since full-length Foxp3 is already in the nucleus when the C-terminal cleavage takes place and the 12-aa tail is released [15].

### The C-Terminal 12-Amino Acids of Foxp3 Contribute to Nuclear Transport (Domain 1)

**Figure 2:**
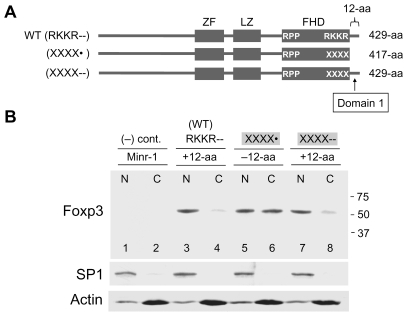
The C-terminal 12-aa residues (Domain 1) contribute to nuclear localization. **A.** Schematic diagram of WT Foxp3 (RKKR--), C-short mutant (XXXX•) and full-length mutant (XXXX--). Domain 1 that corresponds to the C-terminal 12-aa tail and necessary for efficient translocation of Foxp3 is marked. **B.** Western blot showing increased nuclear translocation of Foxp3 following the addition of tail residues (lanes 5–8; −12-aa versus +12-aa). CD4+ cells were harvested 24 hr after retroviral transduction. Lower panels, SP1 (nuclear) and Actin (cytoplasmic) controls.

The differences in the nuclear translocation rates of full-length Foxp3 mutants (RKKX--, XKKX-- and RXXR--) and the C-short mutant (XXXX•) suggested that the C-terminal tail may have a role in nuclear transport of Foxp3 (Fig. 1). To determine whether this is the case, a construct encoding a full-length Foxp3 mutant (XXXX--) was made by attaching a DNA-fragment encoding the 12-aa tail sequences to the C-terminal end of the C-short Foxp3 mutant (XXXX•). The nuclear and cytoplasmic distribution of the short and the full-length Foxp3 mutants (XXXX• and XXXX--) showed the attachment of the 12-aa tail dramatically improved the rate nuclear translocation (Fig. 2, lanes 5 and 6 versus lanes 7 and 8). The result indicates the C-terminal 12-aa tail (14-aa in the human), referred to as Domain 1, contribute to nuclear transport of Foxp3 (Fig. 2).

### Sequences Immediately N-Terminal to the FHD Contribute to Nuclear Transport of Foxp3 (Domain 2)

**Figure 3:**
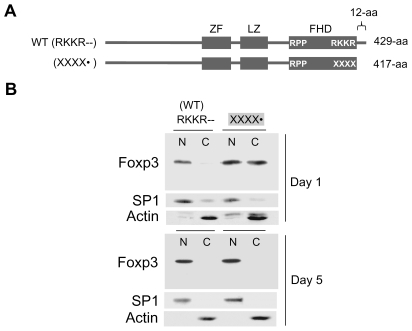
Foxp3 missing Domain 1 and the RKKR residues can fully translocate to the nucleus over time. **A.** Schematic diagram of WT Foxp3 (RKKR--) and C-short Foxp3 mutant (XXXX•). **B.** Western blot showing nuclear and cytoplasmic distribution of WT-Foxp3 and C-short Foxp3 mutant at Day 1 and Day 5 following retroviral transduction. Lower panels, SP1 and Actin controls.

**Figure 4:**
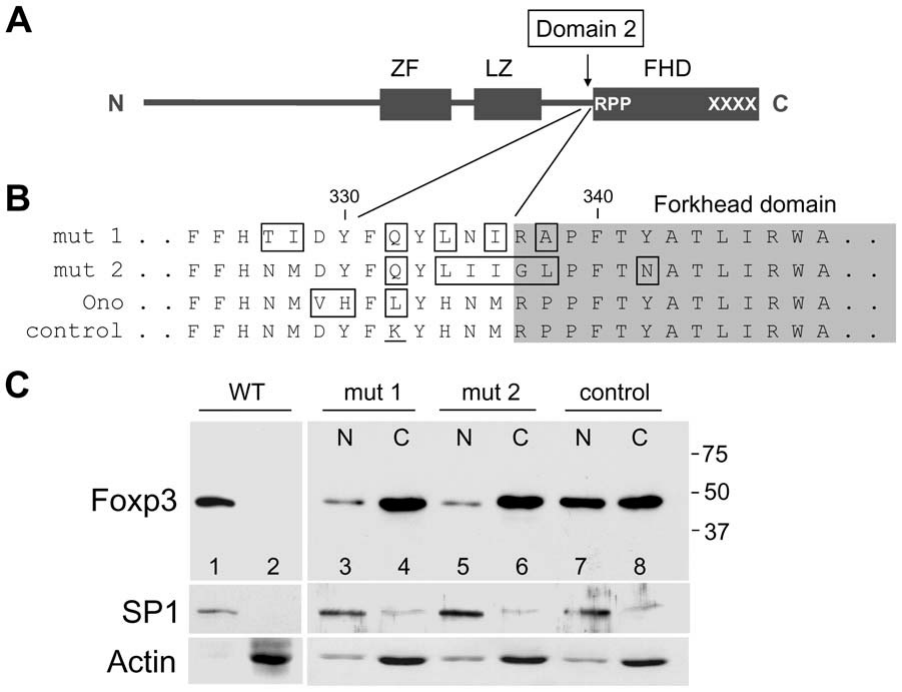
Sequences immediately N-terminal to the FHD (Domain 2) contribute to nuclear translocation of Foxp3. **A.** Schematic diagram of C-short Foxp3 mutant (XXXX•). Additional mutations were introduced in order to identify the second domain that contributes to Foxp3 nuclear translocation. The arrow marks the location of Domain 2 and the converging lines lead to the sequences shown in B. **B.** Mutations in Domain 2 that impair the nuclear translocation ability of Foxp3. Mutant residues in mut 1, mut 2 of this study and of Ono (detailed sequence shown in Ono et al. [9]) are boxed. The 16^th^ lysine in the control C-short mutant is underlined. **C.** Western blot showing the impaired nuclear translocation ability of mutants 1 and 2 (lanes 3, 4 and 5, 6 versus lanes 7, 8). CD4+ cells were harvested one day after retroviral transduction. WT Foxp3 (lanes 1, 2) is from the same experiment but analyzed on a separate blot. Lower panels, SP1 and Actin controls. Abbreviation: mut designates mutant.

The removal of Domain 1 (C-terminal tail) from Foxp3 only slows down the rate of nuclear translocation, and transport is complete by day 5 following retroviral transduction (Fig. 3), indicating additional region(s) in Foxp3 also contribute to its nuclear transport. To identify these region(s), a search was initiated and the C-short Foxp3 mutant (XXXX•) which does not have the RKKR sequence and the C-terminal Domain 1 was further mutated at multiple sites. Mutations in several regions of Foxp3 did not alter the kinetics of nuclear translocation (data not shown). However, a region located immediately N-terminal to the FHD having two histidine-asparagine-methionine (HNM) repeats was found to be important. The HNM repeats, conserved between mouse and human, are separated by a 5-aa spacer region, and the 16^th^ lysine residue of Foxp3 is located within this spacer. The two constructs that pin-pointed this region have partially overlapping mutations. The rate of nuclear translocation of the two mutants (Fig. 4B, mutants 1 and 2; mutant residues boxed) were compared to C-short Foxp3 mutant (XXXX•), used as control. While 50% of the control was found in the nucleus at 24-hrs after retroviral transduction, both mutants 1 and 2, having mutations at approximately the same location, were significantly impaired in their nuclear translocation ability (Fig. 4C, lanes 3–6). Thus, the region immediately N-terminal to the FHD contributes to nuclear transport of Foxp3 and is referred to as Domain 2. It should be noted that the 16^th^ lysine of Foxp3 (residue 332) plays a critical role since when this lysine is kept intact, nuclear transport is only minimally affected (data not shown). This region that includes the 16^th^ lysine was recently reported to be essential for binding of Foxp3 to Runx1 transcription factor [9].

### N-Terminal Sequences of Foxp3 (the First 51-aa) Contribute to Nuclear Transport (Domain 3)

**Figure 5:**
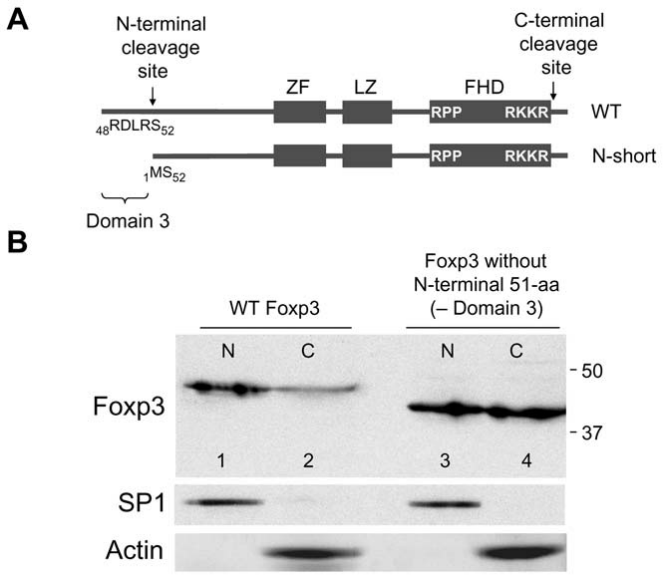
N-terminal 51-aa of Foxp3 (Domain 3) contribute to its nuclear translocation. **A.** Schematic diagram of WT Foxp3 and of Foxp3 missing the N-terminal 51-aa (N-short). **B.** Western blot showing the nuclear and cytoplasmic distribution of WT and N-short Foxp3. The reduced size of N-short Foxp3 (41-kDa) in lanes 3 and 4 is due to the absence of N-terminal 51-aa. CD4+ cells were harvested 20 hr after transduction. Lower panels, SP1 and Actin controls.

**Figure 6:**
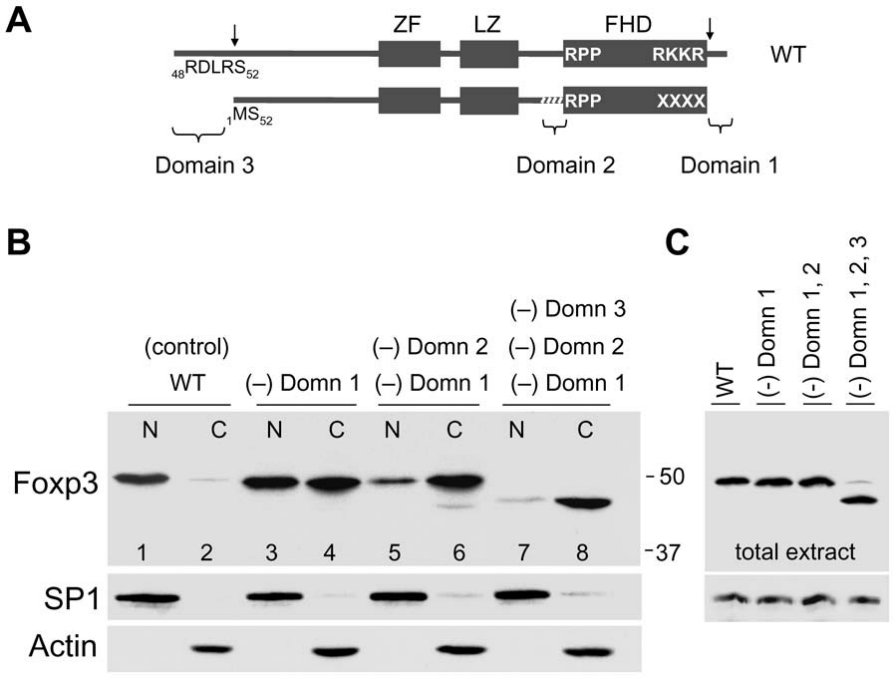
Contribution of Domains 1, 2 and 3 to nuclear translocation. **A.** Schematic diagram of WT Foxp3 and of Foxp3 mutant that lacks the N-terminal 51-aa, Domain 1 and a functional Domain 2 (marked with white slanted lines). Vertical arrows indicate sites of proteolytic cleavage. **B.** Nuclear and cytoplasmic distribution of Foxp3 mutants (XXXX): missing only Domain 1 (lanes 3 and 4); missing Domains 1 and 2 (lanes 5 and 6); missing Domains 1, 2 and 3 (lanes 7 and 8). CD4+ cells were harvested 24 hr after transduction. Lower panels, SP1 and Actin controls. **C.** Western blot showing equal Foxp3 expression in total extracts obtained from samples shown in B. SP1 signal was used to determine loading. Abbreviation: Domn designates Domain.

Earlier experiments comparing the nuclear translocation rates of full-length Foxp3 and an engineered Foxp3 that mimics the N-terminally cleaved form (N-short Foxp3: 378-aa) showed the lack of the N-terminal 51-aa residues reduce the rate of nuclear translocation (Fig. 5B). The role of the N-terminal domain was further probed by studying the nuclear transport of a Foxp3 mutant that lacks the N-terminal 51-aa, Domain 1 and lacks a functional Domain 2. The results show this mutant protein missing Domains 1, 2, and 3 is found almost exclusively in the cytoplasm (Fig. 6B, lanes 7 and 8). The detection of trace levels of this mutant in the nuclear extract (Fig. 6B, lane 7) is likely due to cross-contamination of nuclear and cytoplasmic fractions during the separation process. Hence, the first 51-aa of Foxp3, referred to as Domain 3, represents the third region that contributes to Foxp3 nuclear transport.

## Discussion

**Figure 7:**
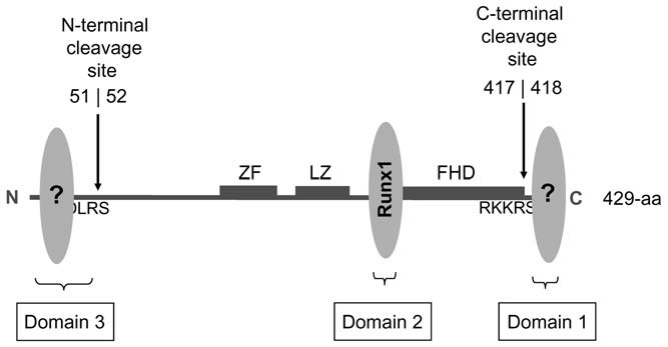
Domains 1, 2 and 3 of Foxp3. Schematic diagram showing the locations of the N-terminal and C-terminal cleavage sites (between residues 51 and 52, and 417 and 418). The approximate locations of Domains 1, 2 and 3 that contribute to nuclear transport of Foxp3 are indicated. Grey oval structures designate factors that bind to these three domains and help nuclear translocation of Foxp3.

This study identifies three domains in Foxp3 that participate in its nuclear translocation. These three domains are located at the C-terminal end (Domain 1), immediately N-terminal to the FHD (Domain 2) and at the N-terminal end of Foxp3 (Domain 3) (Fig. 7). None of these domains are rich in basic residues and do not have similarities to previously described monopartite or bipartite NLSs [21]–[23]. The central domain (Domain 2), located at the N-terminal side of FHD, was recently reported to be a binding site for the transcription factor Runx1 [9]. Thus, it is likely that the other two domains of Foxp3 also interact with nuclear factors that help transport Foxp3 to the nucleus. A similar type of nuclear transport, by “piggy-backing”, has been described for Cyclin B1. Cyclin B1 lacks a NLS but is able to make it to the nucleus by binding to Cyclin F which has multiple functional NLSs [24].

Processing of Foxp3 results in N-terminally or C-terminally cleaved forms of the protein. It is noteworthy that the nuclear factors that dock onto Domains 1 and 3 would likely be released together with the N- or C-terminal ends following proteolytic cleavage. Whether Foxp3 is N-terminally or C-terminally cleaved may determine which factor(s) remain associated with it. Such a mechanism can give Foxp3 the ability to associate with different factors in order to achieve its multifunctional role in transcriptional regulation. The existence of a similar mechanism, the interaction of human Foxp3 with the transcription factors RORα and RORγt, has recently been described. Full-length human Foxp3 (Foxp3a) can bind both RORα and RORγt, whereas the shorter Foxp3b isoform, lacking the exon 2 residues that interact with RORα and RORγt, cannot [10], [11].

Proteolytic cleavage of Foxp3 at the C-terminal RXXR motif (^414^RKKR↓S^418^), located only 12-aa from the C-terminal end, leads to a slightly shorter protein but affects Treg function dramatically [15]. RKKR is reported to be a NLS for several proteins, including for a GFP-Foxp3 hybrid [17]–[20]. This study undertakes a detailed mutational analysis of native full-length Foxp3 to determine whether RKKR functions as a NLS. The results show, RKKR does not function as a NLS in the native full-length protein, possibly due to being masked by the neighboring C-terminal 12 amino acid tail. It is noted that the tail sequence is moderately rich in proline residues, known to introduce kinks and tight turns in proteins [25], [26]. There are 3 prolines in the 12-aa tail of mouse Foxp3 and 4 prolines in the 14-aa tail of the human Foxp3. Thus, proline induced turns in the 12-aa tail may result in concealing RKKR and preventing it from functioning in NLS-mediated nuclear import. In addition, the fact that no RKKR mutations in IPEX patients have been linked to a deficiency in nuclear localization also argues against a NLS role for RKKR. The discrepancy between the results presented here and of Lopes et al., on the role of the RKKR as a NLS, can be explained by their use of a GFP-Foxp3 hybrid instead of the native protein [20]. GFP has a considerable size (26 kDa) which can affect folding of the Foxp3 portion. That even minor deviations from the native structure may have profound functional consequences is supported by the fact that the C-terminal processing of Foxp3, and subsequent deletion of the C-terminal tail which is considered to be a non-critical region for Foxp3, dramatically affects the overall function of the protein [15].

Foxp3 forms a large complex (∼600 kDa) with several factors, including the histone acetyltransferase Tip60, histone deacetylase HDAC7, RORα, RORγt, Runx1 and NFAT [8], [27]. However, neither Domain 1 nor 3 of Foxp3 have been implicated in binding to these factors. Identification of the factors that interact with these domains will not only facilitate our understanding of how Foxp3 achieves its nuclear transport to regulate the expression of target genes, but may lead to development of therapies that modify Treg function.

## Materials and Methods

### Animals

Female C57BL/6 mice (6–8 weeks old) were purchased from The Jackson Laboratory (Bar Harbor, ME). All studies were performed with a protocol approved by the institutional animal care and use committee of The Children's Hospital of Philadelphia.

### Antibodies

Anti-mouse Foxp3 mAb FJK-16s (ebioscience; catalog number 14-5773-82), SP1 and Actin antibodies (Santa Cruz Biotechnology; catalog numbers sc-59 and sc-1615) were used for Western blots.

### cDNA Cloning and Mutagenesis

mFoxp3 cDNA was amplified from thymus with Foxp3-specific forward and reverse primers (Integrated DNA Technologies) and cloned into the Bluescript vector pPCR-Script (Stratagene; catalog number 211188-5). The mutations were introduced with a QuikChange site II-directed mutagenesis kit (Stratagene; catalog number 200523-5). For equal expression, all constructs had the same length of Foxp3 cDNA but were engineered to encode different Foxp3 forms. The different Foxp3 forms were generated either by insertion of a stop codon (for C-short Foxp3) or an initiation codon (encoding methionine; for N-short Foxp3) in the correct locations of the cDNA. Construction of the plasmid that encodes N-short Foxp3 required multiple mutagenesis steps to abolish the natural ATG and another ATG 25-base downstream of the first one, to prevent chain initiation at undesired locations. After sequence confirmation, fragments were recloned to murine stem cell virus (MSCV)-based Minr-1 vector for retroviral expression.

### Retroviral Expression

Retroviruses were generated by co-transfection of WT or mutant Foxp3 (in Minr-1 vector) with pCLeco (Invitrogen) helper plasmid into the 293T-based Phoenix ecotropic packaging cell line, using Lipofectamine 2000 Reagent (Invitrogen; catalog number 52887). Virus containing supernatant was collected and used to infect purified CD4+ cells. Prior to infection with the retrovirus, CD4+ T cells were isolated by magnetic sorting, activated with PMA (3 ng/ml), ionomycin (1 mM) and IL-2 (5 U/ml) for 24 hr, washed, and transduced using the 48 hr viral supernatants obtained from transfected Phoenix cells [16].

### Nuclear and Cytoplasmic Extraction and Western Blots

Following retroviral transduction, CD4+ cells were expanded for either 20 hr, 24 hr or 5 days before subcellular fractionation [28]. Proteins were separated by 14% SDS-PAGE, blotted onto PVDF membrane, probed with the primary Ab followed by secondary Ab-HRP. Signal was obtained with the Luminol reagent (Santa Cruz Biotech; catalog number sc-2048) and recorded onto Kodak MR Film. Prior to Actin antibody treatment, some blots were stripped with the Restore Western Blot Stripping Buffer (Pierce; catalog number 21059) to remove the Foxp3 antibody from the membrane.
